# Notes and Notices

**Published:** 1888-08

**Authors:** 


### NOTES AND NOTICES.

Removals.—After June 1st Dr. Prosper Bender's office will be at 134 Boylston Street, Boston. During July and August the Doctor will be at Atlantic House, Nantucket Beach. Dr. James E. Lilienthal has removed his office to 1316 Van Ness Avenue, San Francisco. Professor S. Lilienthal will also have an office there for consultations.

Atlantic City.—Dr. G. W. Crosby has associated with him in his practice Dr. G. A. Rathburn.

The Missouri Hom. Med. College has chosen Dr. W. L. Reed to succeed Dr. J. T. Kent in the chair of Materia Medica and Therapeutics. The choice is considered a good one, and it is believed that Dr. Reed will continue the work in the College so well begun by Dr. Kent.

Errata.—In the very interesting report of a case of Retinitis Pigmentosa, given in our June issue, by Dr. Fredk. W. Payne, of Boston, *Arum* was written for *Aurum* (see p. 290, lines 6 and 10). In the lecture upon Podophyllum, p, 282, line 18, read *Prolapsus of rectum when urinating, Mur-ac.* In the text *vomiting* was given for *urinating.* In our May issue, p. 243, line 12, from bottom, Dr. Biegler is reported as giving *ten* remedies in a case where *two* cured.

Another Medical School.—A medical department has been added to the Minnesota State University. It is to be divided into three schools—one for surgery and medicine ("regular"), another for Homœopathy, and a third for dentistry.

The faculty of the College of Homœopathy is as follows: Theory and Practice, Dr. Henry Hutchinson, St. Paul; Materia Medica and Therapeutics, Dr. W. E. Leonard, Minneapolis; Obstetrics. Dr. H. C. Leonard, Fergus Falls; Gynæcology, Dr. A. E. Higbee, Minneapolis; Principles and Practice of Surgery, Dr. R. D. Matchan, Minneapolis; Pædology, Dr. H. W. Brazie, Minneapolis; Clinical Medicine, Dr. Geo. E. Ricker, Minneapolis; Ophthalmology, Dr. J. F. Beaumont, Minneapolis. Lectures: Clinical Surgery, Dr. W. S. Briggs, St. Paul; on Dermatology, Dr. H. C. Aldrich, Minneapolis; on Physical Diagnosis and Laryngology, Dr. E. L. Mann, St. Paul; on Nervous Diseases, Dr. S. M. Spalding, Minneapolis; on Genito-urinary Diseases, Dr. H. B. Ogden, St. Paul; on Otology, Dr. D. A. Strickler, Duluth.

Dr. E. A. Ballard, of Chicago, recently paid a visit to our sanctum on his return from the I. H. A. meeting at Niagara. In course of conversation he gave us these therapeutic hints. "In the provings of Cyclamen there is no mention of aggravation *from heat.* Yet I have relieved a case of hay fever where this condition was prominent. Every warm day the patient became worse; she was especially worse going into the kitchen near the fire. Cyclamen has promptly controlled this case, both this and last season. One hot day recently the patient spent the day near her kitchen fire, ironing, and yet she suffered no return of sneezing or other symptoms."

A Post-Graduate Course of Lectures.—A post-graduate course of lectures will be given under the auspices of the Executive Board of the Women's Homœopathic Association of Pennsylvania, at the Hospital, Twentieth Street and Susquehanna Avenue, Philadelphia.

Professor J. T. Kent will deliver lectures on Homœopathic Materia Medica and the *Organon* on Tuesday, Wednesday, Thursday, and Friday afternoons, from half-past five to half-past six o'clock, commencing October 16th and continuing seven weeks. Course tickets, forty dollars. For special course on *Organon*, twenty dollars, and on Materia Medica, twenty dollars.

Tickets may be procured from Mrs. M. T. Keehmle, Treasurer of the Association, 1315 Arch Street, Philadelphia.

American Institute held its annual meeting at Niagara Falls, June 25–29. Dr. Cowperthwaite, the President, delivered a good address, better than the usual, and the attendance was far above the average. The work done was about the usual routine work of the Institute. In the last two or three years the Institute seems to be giving a little more attention to the *Materia Medica* than has been its custom for many years, but even now pathology, surgery, and kindred subjects engross too much of its time and labor. The usual exhibit of surgical instruments, of quack drugs, etc., was on hand. In old-time Institute days, such things were not seen, for the physicians of that day did not *need* such adjuvants. They would have considered themselves insulted had they been exhibited! Dr. Selden H. Talcott was elected President, and Lake Minnetonka, Minn., selected as the place for the next meeting.

